# Novel Hydrogels for Topical Applications: An Updated Comprehensive Review Based on Source

**DOI:** 10.3390/gels8030174

**Published:** 2022-03-10

**Authors:** Yosif Almoshari

**Affiliations:** Department of Pharmaceutics, College of Pharmacy, Jazan University, Jazan 45142, Saudi Arabia; yalmoshari@jazanu.edu.sa

**Keywords:** hydrogel, novel formulations, natural polymer, synthetic polymer, topical application

## Abstract

Active pharmaceutical ingredients (API) or drugs are normally not delivered as pure chemical substances (for the prevention or the treatment of any diseases). APIs are still generally administered in prepared formulations, also known as dosage forms. Topical administration is widely used to deliver therapeutic agents locally because it is convenient and cost-effective. Since earlier civilizations, several types of topical semi-solid dosage forms have been commonly used in healthcare society to treat various skin diseases. A topical drug delivery system is designed primarily to treat local diseases by applying therapeutic agents to surface level parts of the body such as the skin, eyes, nose, and vaginal cavity. Nowadays, novel semi-solids can be used safely in pediatrics, geriatrics, and pregnant women without the possibility of causing any allergy reactions. The novel hydrogels are being used in a wide range of applications. At first, numerous hydrogel research studies were carried out by simply adding various APIs in pure form or dissolved in various solvents to the prepared hydrogel base. However, numerous research articles on novel hydrogels have been published in the last five to ten years. It is expected that novel hydrogels will be capable of controlling the APIs release pattern. Novel hydrogels are made up of novel formulations such as nanoparticles, nanoemulsions, microemulsions, liposomes, self-nano emulsifying drug delivery systems, cubosomes, and so on. This review focus on some novel formulations incorporated in the hydrogel prepared with natural and synthetic polymers.

## 1. Introduction

Generally, active pharmaceutical ingredients (API) or drugs are not supplied as pure chemical compounds (for the prevention or the treatment of any diseases). Still, APIs are commonly delivered in pharmaceutical formulations, commonly known as dosage forms [1]. It is a multistep process that involves the combination of API with other components widely known as excipients or pharmaceutical inactive ingredients and converting into a final valuable medicinal compound [2]. They have been converted into a suitable dosage formulation and delivered in various administration routes [1]. The dosage forms are commonly administered in solid, semi-solid, and liquid forms. The various solid dosage forms include tablets, capsules, and powders. The various semi-solid dosage forms include creams, gel, emulgel, ointments, paste, etc. The various types of liquid dosage forms include emulsion, injections, lotion, ocular formulations, suspension, syrup, etc. [3].

For delivering therapeutic agents locally, topical administration is mainly preferred due to its convenience and affordability. A topical drug delivery system (TDDS) is mainly meant for treating local diseases by applying the therapeutic agents to superficial body parts, including the skin, eyes, nose, and vaginal cavity. In contrast to oral administration, topical administration avoids first-pass metabolism in the liver, gastric pH variations in the stomach, and fluctuations in plasma concentrations. The other benefits of a topical drug delivery system consist of patient fulfilment and acceptance, easy and convenient application, less pain and noninvasive system, increase in API bioavailability, improved physiological and pharmacological action, as well as minimal systemic toxicity and exposure of API to non-infectious tissue/sites [4].

The production of semi-solid products has been going on for decades, and they are often used as pharmaceuticals, cosmetics, and health supplements. Typically, semi-solid dosage form (SSDF) products are administered topically or inserted into a body orifice [5]. This dosage form does have some benefits, such as applying the medications directly to the affected area and the ease of administration to patients of any age. A challenge in SSDFs is the need to deliver the API across the skin or other physical membranes of the patient to reach the desired system [6]. Since ancient times, there have been several kinds of topical semi-solid dosage forms widely used in human society for treating various skin diseases [7]. The semi-solid dosage forms used topically usually come in creams, gels, ointments, or pastes [8]. Dermatology products are available in various formulations and consistencies, but semi-solid dosage forms are the most popular kind [9].

Skin is the most important and largest organ in the body. It is responsible for a variety of necessary functions, mainly protection (from an entry of microorganisms, from the external environment, to prevent excessive water loss, etc.,) as well as to regulate body temperature [10,11]. In TDDS, skin is one of the primary and accessible organs [9]. Most topical semi-solid formulations are designed to target the skin or underlying tissues [7]. Among the three layers (stratum corneum (SC), epidermis, and dermis) of skin, the SC is primarily responsible for protecting the tissues underneath the skin. Most of the API’s cannot easily pass through the skin due to the effective barrier of the SC [10]. The SC restricts the penetration of nearly all large and hydrophilic API molecules, including proteins, peptides, nucleotides, and oligonucleotides [12].

Despite their washable water bases, novel semi-solids are not greasy. Therefore, they are less irritating to the skin and more effective than conventional semi-solid dosage forms. Novel semi-solids can be used safely in pediatrics, geriatrics, and pregnant women without the possibility of causing any allergy reactions. It is expected that novel semi-solids will control the release pattern [13]. Topical dosage forms that are intended to give local or systemic effects. Conventional TDDS has significant drawbacks. Acne, alopecia, and psoriasis are all skin diseases with deep roots inside the skin. Traditional TDDS seems inadequate in treating the above skin disease due to the poor absorption of APIs in the skin.

Due to skin’s poor retention, conventional topical dosage forms appear ineffective in treating these conditions. Based on patient fulfillment, security, effectiveness, feasibility, and shelf life, novel TDDS has gained popularity in recent decades. Based on the research results performed in novel TDDS, it can be concluded that transferring from conventional TDDS to novel TDDS through the use of carriers necessitates extensive research and will offer new hope for the treatment of various diseases [9].

According to the USP, gels (also known as jellies) are semi-solid systems made up of either a suspension containing small inorganic particles or organic macromolecules (primarily polymers) dissolved in a large quantity of liquid to form an infinite rigid network structure [14]. According to Rathod and Mehta [15] and also by Jeganath and Jeevitha [16], there are several ways to classify gels, including colloidal phases (inorganic and organic), solvents type (emulgel, hydrogels, organogels, and xerogels), composition (flexible and inflexible gels), and rheological property (plastic, pseudoplastic, and thixotropic gels). According to Paul et al. [17], the novel formulation can include into the following gels: (i) hydrogels, (ii) organogels, (iii) in situ gels, (iv) emulgels, (v) microgels, (vi) nanogels, and (vii) vesicular gels (liposomal gel, niosomal gel, and transferosomal gel).

A semi-solid state became advantageous for actual usage of the prescribed dose for applying in the skin. Pharmaceutical companies are gaining rapid attention or are increasingly interested in hydrogels due to their modified drug release [18]. Initially, many research studies were performed in hydrogels by simply incorporating various drugs in pure or dissolved in suitable solvents to the prepared hydrogel base. However, for the past five to ten years, numerous research articles on novel hydrogels have been published. Novel hydrogels are made up of novel formulations such as nanoparticles, nanoemulsions, microemulsions, liposomes, self-nano emulsifying drug delivery systems, cubosomes, and so on. The novel hydrogels are used for different applications.

Few review articles related to novel hydrogels have been previously published in various journals. Torres et al. [19] reviewed classification of hydrogels based on physical or chemical interactions as well as stimuli-sensitive substances. Taxol (a Paclitaxel loaded formulation) loaded with biocompatible nanocarriers (nanoparticles, microparticles, micelles, liposomes, etc.) incorporated into hydrogels prepared with natural polymers (hyaluronic acid, gellan gum, alginate, and chitosan) was reviewed by Voci et al. [20]. A chitosan and cellulose-based hydrogel for wound treatment was reviewed by Alven and Aderibigbe [21]. Michalik and Wandzik [22] reviewed chitosan-based hydrogel in the agriculture field for sustained action. Cai et al. [23] reviewed and discussed the latest findings in advanced hybrid-based hydrogel technologies combining nanostructures and microstructures, their formulation, and potential uses mainly in tissue engineering and antitumor delivery systems. The current review aims to highlight the most important developments that have come about from the increase of a variety of novel formulations containing the API embedded in different sources of polymer-based hydrogels. This review article discusses some previous research on topical novel hydrogels prepared with varying sources like natural and synthetic gelling agents.

## 2. Methodology

To obtain the appropriate literature, relevant keywords like hydrogels, topical hydrogels, novel topical hydrogels, etc. have been used in search engines such as Google Scholar, PubMed, etc. The information was mainly collected from research and review articles published between 2010 to 2022.

## 3. Hydrogels

Hydrogel refers to a three-dimensional arrangement made of hydrophilic polymers with a high capacity to interact with and retain a vast amount of water and biological fluids due to several functional grouping [like amino (–NH_2_), the carboxylic acid (–COOH), a hydroxyl group (–OH), amide (–CONH), sulfo groups (–SO_3_H)] in the polymer chains [24]. As per Lee, Kwon, and Park, the term “hydrogel” comes from an article published in 1894 and the first cross-linked network substance that showed up in publications [25]. In 1960, Wichterle O from Prague and Lim D from the Czech Republic were the first to discover hydrogels. They used synthetic polymer poly-2-hydroxyethyl methacrylate for preparing contact lenses [4,26]. Due to the high-water holding capacity, elastic in nature, compatibility with living tissue, and adaptability made hydrogels as a broad choice of use in various fields [27]. As part of the second stage, commencement in the 1970s, scientists began developing a more complex hydrogel capable of responding to pH and temperature and producing a precise response. Third-stage hydrogels are made from supramolecular inclusion complexes that are biocompatible and versatile. Hydrogels developed in the third stage of development led to creating “smart hydrogels” [28]. Since the 1980s, hydrogels have been used for various biomedical disciplines like contact lenses, absorbent cotton, suture, and cell engineering [29], and biosensor, drug delivery, cell therapy, and 3D cell culture [27] to incorporate various conventional and novel formulations [18,29].

## 4. Classifications of Hydrogels

**Figure 1:**
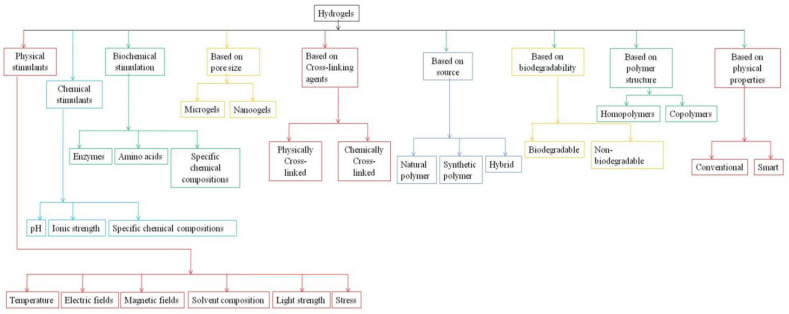
Classification of hydrogel.

Hydrogels can be classified into multiple ways with various viewpoints based on the literature. Based on phase transition like gel-sol reactions due to physical or chemical, or biochemical stimulation. The physical stimulants comprise temperature, electric fields, magnetic fields, solvent composition, light strength, and stress. In contrast, chemical stimulation includes pH, ionic strength, specific chemical compositions, and biochemical stimulation by enzymes and amino acids [24]. Based on their pore size (nanogels and microgels), based on the polymer structure (homopolymers and copolymers), based on cross-linking (physically cross-linked and chemically cross-linked), based on degradation (biodegradable, non-degradable) and their source (natural, synthetic, or hybrid), and based on physical properties (conventional, and smart) [20], the classification of hydrogel is shown in Figure 1.

Hydrogels provide comfortable drug delivery methods because of their tunable properties, rapid expandable degradation, and attainable preparation. [30]. A hydrogel is an excellent carrier for delivering APIs, especially to the skin. Ex vivo porcine ear skin is a widely used model due to its similarity to the human skin in terms of behavior, composition, and permeability [31].

## 5. Hydrogels Prepared with Natural Polymers

Natural hydrogels were prepared with natural constituents recommended for maximum biocompatibility as they are obtained from natural sources [28]. Novel formulation formulated with natural gelling agents, preparation methods, mixing with the hydrogels, and its use are shown in Table 1. Different novel formulation evaluation studies before incorporating into hydrogel are shown in Table 2. Physical evaluation studies for novel natural hydrogel formulation prepared with natural gelling agent are shown in Table 3. Novel natural hydrogel formulation in vitro, in vivo evaluation studies details are shown in Table 4.

### 5.1. Hydrogels Loaded with Liposomes

Liposomes (LS) are nanocarriers primarily composed of phospholipids and cholesterol [42]. *Chlamydia trachomatis* is the causative agent of sexually transmitted infections. The current treatment option is oral azithromycin or doxycycline, both of which have potential side effects. To reduce the side effect and effectively treat *C. trachomatis*, Jraholmen et al. [32] used natural polyphenol Resveratrol (RVT) LS incorporated into a natural CHI hydrogel. To maximize RVT’s potential therapeutic activity, they used LS as the primary release medium and CHI hydrogel as a supplemental medium. Since RVT inhibits biofilm formation, LS are preferred nanocarriers for topical therapy since it does not interact with vaginal flora, and chitosan hydrogel effectively obstructs vaginal biofilms. RVT in LS preparation was found to increase RVT solubility, deliver sustained action, and enhance chemical stability, allowing for medical uses. In addition, RVT in LS formulation improves RVT’s ability to bind to microbes, producing a more potent antimicrobial action even at a low dosage. The study showed that RVT-LS in CHI hydrogel could inhibit nitric oxide, the chief free radical that causes inflammation. The RVT-LS incorporated into the CHI hydrogel delivery system improved the anti-chlamydial effect of RVT at lower concentrations and highlighted the existence of a delivery system to ensure effective treatment.

### 5.2. Hydrogels Loaded with Self-Double-Emulsifying Drug Delivery System

Self-double-emulsifying drug delivery system (SDEDDS) is a mix ture of water-soluble surfactants and water-in-oil (w/o) emulsions, which can instantaneously emulsify to water-in-oil-in-water (w/o/w) double emulsions, with water-soluble drugs present in the internal aqueous phase [43]. Vitamin C is one of the most effective antioxidants for preventing skin damage and a whitening agent. Once exposed to oxygen, basic pH, and high temperatures, vitamin C is highly unsteady, rapidly degraded, and discolored. As a result, a promising strategy for resolving these weaknesses is required to enable its clinical implementation. To overcome the above and improve the vitamin C penetration in the skin, Wang Q et al. [33] prepared a vitamin C-loaded SDEDDS, followed by blending it in a xanthan gum (XG) hydrogel. Vitamin C-loaded SDEDDS showed improved physical potency upon incorporation into hydrogels, implying that the shell is more secure to protect vitamin C from degradation, mainly from ionization solution and oxygen exposure. Incorporating vitamin C in SEDDS-based hydrogels increases the vitamin C permeation in the skin due to the bioadhesive property of XG. The oil vesicle of the SDEDDS could even act as a protective coating for vitamin C, allowing for improved vitamin C-controlled release from the SDEDDS formulation. Vitamin C encapsulation in SDEDDS, hydrogels, or even both might greatly increase vitamin C permeability and distribution inside the skin. Overall, vitamin C-incorporated SDEDDS-mixed with XG hydrogels will greatly enhance skin penetration.

### 5.3. Hydrogels Loaded with Microparticles

Polymeric microparticles (MP) have been extensively researched as a beneficial and novel carrier for the sustained and controlled release of vast drugs. Chronic wound healing treatment usually requires the administration of drugs at regular intervals for a more extended period. Long-term sustained release treatment might decrease the frequency of administration while maintaining drug concentration at the site of a wound. Based on this knowledge, Yasasvini S et al. [44] created simvastatin (SIM) CHI CHI-MP and incorporated them into polyvinyl alcohol (PVA) hydrogels to improve wound healing activity. After seven days, 92% of SIM was released from 5% PVA hydrogel for a dose of 2.5 mg. This SIM release from 5% PVA was correlated with the swelling index. In low dose (2.5 mg), the swelling index value was more when compared to 5 and 10 mg SIM concentration. The in vivo wound healing activity showed SIM released in controlled manner results in continuous wound healing. The combination of APIs in MP formulation incorporated into hydrogels could be best for releasing the APIs in a sustained manner and a successful topical wound healing activity from the above results.

### 5.4. Hydrogels Loaded with Nanoemulsion

Nanoemulsions (NE) are thermodynamically stable and spherical structures where a thin layer of emulsifying agent stabilizes the oil droplets that contain the drug. NE is getting popular for enhancing the skin permeation of lipophilic drugs [45]. Rosmarinic acid (ROS) is a natural polyphenol. Due to its antioxidant property, it can be used as anti-aging compound. But its use is limited due to poor water solubility, high instability, and poor permeability through biological barriers. To overcome the above drawbacks Marafon et al. [34] created ROS loaded NE for topical use. The pH of the ROS-NE was ranged from 3.80 to 5.80, making the formulation physically stable and suitable for topical application. The permeability studies in porcine ear skin showed that nearly 1.5 fold higher ROS was penetrated when compared to pure ROS. The absence of necrosis in keratinocyte cells indicates the formulation’s safety. Their findings showed that ROS as a NE prepared with tween-80 and incorporated in HEC-based gelling agents could be used as an appropriate carrier for skin application in novel anti-aging skincare preparation.

Another example for NE encapsulated hydrogel formulation was prepared with Pentyl Gallate (PG) by Kelmann RG and colleagues [35]. PG-NE was prepared and encapsulated into a hydrogel formulation containing natural polymer CHI and a synthetic polymer Aristoflex AVC (ART). PG-NE-ART’s viscosity is less than PG-NE-CHI; this increase in viscosity is due to the degree of deacetylation of CHI. The pH of both the PG-NE-CHI and PG-NE-ART was reduced after 90 days due to the hydrolysis of the triglyceride and phospholipid moieties, which resulted in the release of free fatty acids. The presence of a CHI or an ART did not affect the skin retention of PG from the NE. ART compared to the two gelling agents tested, ART appeared to be more intriguing since it initially did not affect PG-NE’s droplet size and zeta potential. The PG-NE-ART also demonstrated improved intrinsic stability, viscosity, and spreadability. According to the authors, PG can be an attractive topical antiherpetic agent.

Phenytoin (PENY) is an antiepileptic drug on prolonged oral administration that results in gingival hyperplasia. Previous studies confirmed that PENY could be used successfully as a wound-healing agent, but none of the studies clearly states the permeation of PENY across the skin. Cardoso et al. [36] prepared PENY-NE and nanocapsule (NC), to which CHI was dispersed and converted into novel hydrogels, and then evaluated PENY skin penetration and wound healing activity. The in vitro release study results showed that PENY diffuses through the NE and NC and then through the hydrogel network to arrive at the release medium in a controlled manner. The higher PENY accumulation in the dermis of injured skin for PENY-NC-CHI and PENY-NE-CHI hydrogels compared to other skin layers suggested that such hydrogels might be helpful to formulations for wound treatment. When PENY-loaded NC and NE hydrogels were compared to the non-encapsulated form, the amount of PENY that reached the dermis and the receptor medium was significantly lower. It indicates that PENY as a wound-healing agent poses a low risk of systemic absorption.

### 5.5. Hydrogels Loaded with Microemulsion

The formation of microemulsion (ME) is straightforward, comprising an oil phase, a surfactant, a cosurfactant, and aqueous phases, and can easily be prepared through gentle stirring. ME is an encouraging nanocarrier for the topical administration of poorly water-soluble drugs [46]. Ibuprofen (IBU) is a non-steroidal anti-inflammatory poor water-soluble drug. The skin restricted its percutaneous penetration from conventional preparations. Nanocarriers play a vital role in enhancing the IBU permeation in the skin. To increase IBU percutaneous delivery, Djekic L et al. [37] created an o/w ME with IBU and incorporated it into hydrogel made up of XG. ME-XG-hydrogel prepared with the least XG (0.25%) had the most excellent drug release rate and spreadability. Artificially stimulated inflammation in male Wistar rats via intraplantar injection of carrageenan dispersed in saline. The ME-XG-H1 formulation produced approximately 65% antihyperalgesic effect and 74% antiedematous after 3 h, showing a considerable lowering in paw inflammation caused by carrageenan. The hydrogel-thickened ME could offer promise carriers with improved percutaneous delivery to prevent pain and, to a lesser extent, inflammation.

Invasive fungal infections pose a significant and growing risk to human healthiness. Terbinafine hydrochloride (TER) is an anti-fungal agent used in topical and oral forms. Celebi et al. [38] prepared TER-ME in which natural gelling agents such as CHI (1%) and natrosol 250 (4%), as well as synthetic gelling agents such as CAR-974 (1%), are dispersed. The anti-fungal activities of the TER-ME-loaded hydrogels were evaluated against a variety of strains. The anti-fungal activity was determined against three yeast species (*Candida albicans*, *Candida krusei*, and *Candida parapsilosis*), two nondermatophytic fungi (*Aspergillus niger* and *Penicillium*), and two dermatophytes (*Microsporum spp*. and *Trichophyton rubrum*). TER loaded in CHI hydrogel was the most effective against *Candida albicans* and *Candida krusei*. TER loaded in natrosol hydrogel was most effective against *Trichophyton rubrum*. Based on the findings of their study, they concluded that TER in CHI, CAR-974, natrosol hydrogel, and microemulsion formulations had similar anti-fungal activity as the marketable product (Lamisil).

Curcumin (CUR) administration via percutaneous route could be appropriate for topical and systemic therapeutic applications. Koop et al. [39] created an ME containing the hydrophobic drug CUR and incorporated it into a hydrogel combining xanthan and galactomannan (X-GAL) for topical use. About <60% of CUR was released from CUR-ME-X-GAL. The skin permeation study was carried out on porcine ear skin, and the results revealed that similar amounts of CUR (2.17 to 2.47 µg/mL) were found in the SC, epidermis, and dermis of the skin. In vivo anti-inflammatory study in male Swiss mice showed the inhibition of inflammation of 63.2% for CUR-ME-X-GAL. The researchers hypothesized that combining CUR’s bioactivity with X-GAL hydrogels aided tissue restoration.

### 5.6. Hydrogel Loaded with Nanocrystals

Nanocrystals (NCY) are colloidal carriers of nano-sized particles stabilized in a dispersion medium by least polymeric and/or amphiphilic stabilizing agents. NCY was designed to enhance the absorption and permeation of lipophilic drugs [47]. Baicalin (BCN) cannot be used in topical applications due to its lipophilicity and poor permeation. Wei S et al. [40] attempted to prepare BCN-NCY and incorporate them into a hyaluronic acid (HA) hydrogel to improve BCN topical permeation. The 6-h in vitro release study revealed that more than 95% of BCN was released from BCN-NCY incorporated into 0.5 and 1% HA, while 85.4 and 72.3% of BCN was released from BCN-NCY incorporated into 1.5 and 2% HA, respectively. Over 12 h, the cumulative amount of BCN that permeated rat skin from BCN-NCY-hydrogel with 0.5 and 1% HA was significantly more significant than that of BCN-NCY hydrogel with 1.5 and 2% HA. This is because the viscosity of 0.5 and 1% HA gels was lower than that of 1.5 and 2% HA hydrogels. The 1% HA is relatively advantageous for preparing excellent BCN-NCY hydrogel. According to their study, HA-based NCY-hydrogel can deliver poorly water-soluble drugs topically, safely, and effectively.

### 5.7. Hydrogel Loaded with Cubosomes

Cubosomes (CUBO) are thermodynamically stable; self-assembled nano-sized liquid crystalline particles prepared by combining a specific ratio of lipids with suitable surfactants, water, and temperature conditions [48,49,50]. Silver sulfadiazine (SSD) helps treat burns, but it has drawbacks such as poor permeation into the burn wound and cytotoxicity. However, permeation can be increased and can reduce SSD cytotoxicity with the help of nanocarriers. Morsi et al. [41] prepared a CUBO based on silver sulfadiazine (SSD). They incorporated it into a CHI (1.5%), CAR 940 (1%), or a combination of CHI-CAR-based hydrogels to create cubosomal hydrogels or cubogel. The release of SSD from SSD-CUBO formulation is controlled; thereby, the cytotoxic effect of silver is avoided. Formulating SSD-CUBO into cubogels using CHI and CAR-934 showed advantages over the marketed product Dermazin. From the first day of treatment, there was no interference in the healing process as well as it being compatible with a biological fluid. Healing tissues started earlier (day 9) for SSD-cubogel than Dermazin (day 15). SSD cubogel could thus be used very effectively in administering deep second-degree burns, resulting in improved healing outcomes with few adverse effects compared to most formulations on the market.

## 6. Hydrogels Prepared with Synthetic Polymers

Synthetic hydrogels are prepared with synthetic gelling agents that seem to be more reproducible, have greater flexibility for adjusting their chemical or mechanical qualities, and have more closely controlled structures. However, synthetic hydrogels cannot offer similar biocompatibility to natural hydrogels [28]. Novel formulation formulated with synthetic gelling agents, preparation methods, mixing with the hydrogels, and its use are shown in Table 5. Different novel formulation evaluation studies before incorporating into hydrogel are shown in Table 6. Physical evaluation studies for novel hydrogel formulation prepared with synthetic gelling agent are shown in Table 7. Novel synthetic hydrogel formulation in vitro, in vivo evaluation studies details are shown in Table 8.

### 6.1. Hydrogels Loaded with Liposomes

Photothermal therapy transmitted by near-infrared light (NIR) has emerged as an attractive cancer cell treatment method and an alternative to conventional tumor therapy. Chen G et al. [51] prepared IR780 iodide (tumor-targeting photosensitizer) and IR792 perchlorate (tumor non-target photosensitizer) LS loaded in hydrogels for systematically targeted cancer photothermal therapy. Images of photosensitizers against CT-26 tumor behavior in Balb/c mice showed that compared to IR792, IR780 resulted in the most significant tumor accumulation and a strong fluorescence in tumor tissue parts for the patches applied to mice’s backs. The IR780-LS in hydrogel could systematically target tumors after topical administration. IR780-LSs applied topically in hydrogel increased lung fluorescence signals, confirming IR780 to be targeted effectively against deep metastases. The anti-cancer effect in CT-26 colon tumor mice showed that IR780-LSs in hydrogel-treated mice showed outstanding anti-cancer activity (which could be attributed to a higher concentration of photosensitizers at the tumor site) are nontoxic under laser irradiation. The hydrogel’s biosafety of the IR780-LS was tested in a mouse skin model. The absence of any skin reaction or toxicity during the seven-day course of therapy indicated that IR780/LP in the hydrogel applied topically were secure for the skin. IR780-LS in hydrogel forms a potent combination formulation for topical administration against tumor cells.

### 6.2. Hydrogels Loaded with Self-Nanoemulsifying Drug Delivery Systems

Self-nanoemulsifying drug delivery systems (SNEDDS) are homogeneous mixtures of API combined with lipids, emulsifiers, and hydrophilic co-solvents/solubilizers, which create nanosize emulsions (generally <50 nm) upon constant mixing in the aqueous phase. SNEDDS have a high capacity to solubilize lipophilic APIs, and they also improve the skin permeation [61]. Cutaneous leishmaniasis (CL) is a significantly ignored tropical skin disease that is the most widespread form of leishmaniasis, evidenced by skin lesions that can lead to ulcers, scars, impairment, and stigma [62]. Liposomal amphotericin B intravenously showed good efficacy, but treating with a topical application is preferred due to it being self-administration and cheaper. For the effective and safe treatment option, CL Lalatsa et al. [52] prepared topical buparvaquone (BQ) SNEDDS-enabled carbopol hydrogels. Treatment with BQ-SNEDDS gel indicates no skin modifications (like inflammatory conditions or skin redness); the epidermis and dermis layers were free of inflammatory cells, with no acanthosis or hyperkeratosis. After seven days, the topical application of BQ-SNEDDS gels reduced the parasite load by 99%, very similar to the intralesional administration of Glucantime. Histology studies confirmed that the Balb/c mice treated with BQ-SNEDDS hydrogels showed a reduction in parasitism and evidence of healing. In conclusion, their findings suggest that nano-enabled BQ hydrogels may offer a safe, non-invasive therapy for CL.

### 6.3. Hydrogels Loaded with Phytosomes

Phytosomes (PHY) are typically made by combining APIs with phospholipids in a precise molar ratio under controlled conditions. PHY are an advanced lipid-based delivery method with a LS-like structure that can entrap various polyphenolic-based phytoconstituents to improve absorption when administered [63]. Topical escin-containing components have conventionally been used to relieve leg discomfort and heaviness caused by mild vascular circulatory disturbances and reduce bruises. However, it was unclear if escin itself could decrease pain hypersensitivity in the inflammatory process. To confirm the anti-inflammatory effect of escin, Djekic, et al. [53] and colleagues created topical hydrogels containing escin-sitosterol phytosomes (ES) and escin for antihyperalgesic activity. ES and escin-incorporated hydrogels had significant, concentration-dependent antihyperalgesic effects in a rat inflammatory pain model. CAR-934 hydrogels containing 1–2% ES can be recognized as potential topical formulations that are highly significant to hydrogels containing the same concentrations of escin. The skin irritation test on male Wistar rat skin demonstrated that it could use it safely on human skin. The above research results might imply that topical monocomponent hydrogels containing ES and escin could effectively treat inflammation-related pain. ES-incorporated hydrogels were considerably more efficient than those incorporated with escin.

### 6.4. Hydrogels Loaded with Nanoparticles

Alginate (ALG) hydrogel holds active compounds such as various drugs, signaling molecules, or stem cells with soft flexible gels in ALG rich in M-blocks and firm gels in ALG in rich G-blocks. A few examples of novel approaches based on alginate hydrogels are: Porous 3D hydrogel calcium alginate (Ca-ALG) has great swelling capacity in wounds, providing slow drug release. It is used to entrap cells for tissue regeneration and engineering, as physical support for cells or tissue, or as a hurdle between two media. It protects the cells from the host’s immune system until it reaches the targeted area. The encapsulated fibroblasts represent an excellent example of a dual-layered structure made from alginate hydrogel with apical keratinocytes. Hydrogel film based on poly (N-vinyl caprolactam)-calcium alginate (PVCL/PV-Ca-ALG) loaded with thrombin receptor agonist peptide has shown a beneficial effect on wound healing and tissue regeneration. A relatively recent study compared a sodium alginate-acacia gum-based hydrogel loaded with zinc oxide nanoparticles (ZnO-NPs) to only ZnO-NPs by their healing effects and activity against *B. cereus* and *P. aeruginosa* [64].

Polymeric nanoparticles (NPT) are colloidal particles that are sub-micron (1 to 1000 nm) in size and contain APIs entrapped inside or adsorbed to the polymer [65]. The 5,10,15,20-tetrakis(1-methylpyridinium-4-yl)-porphyrin tetra-iodide (TMPTI) is a well-known porphyrin that is broadly used for the inactivation of different types of microorganisms. TMPTI, on the other hand, is not widely used due to accumulation in healthy cells. Nanoparticles can rectify it; by keeping this concept, Gonzalez et al. [31], with his research team, synthesized TMPTI-NPT by a solvent evaporation method encapsulated into CAR hydrogel. A skin permeability study in domestic porcine skin showed a better TMPTI permeated deeper skin levels with no surrounding damages. It shows that the TMPTI-NPT encapsulated in hydrogel formulation does not affect the normal cells. These findings indicate that encapsulating topical pharmaceutical carriers like hydrogels may successfully treat topical skin diseases, including skin cancer.

Solid lipid nanoparticles (SLNP) are made up of solid lipid compounds, incorporated with an API in them, and coated with a surfactant to stabilize their structure [66]. Articaine (ATC) is a local anesthetic drug, but continuous usage leads to prolonged and permanent paresthesia. Melo et al. [58] aimed to create NC and SLNP containing ART, which was used topically by incorporating ART hydrogels attained a sustained release of ATC from the NC and SLNP formulation. The encapsulation of ATC in NC and SLNP results in higher cellular viability values. The formulation containing ATC-NC showed the best permeation characteristics compared to SLNP. This study opens up possibilities for the future use of nanostructured delivery systems for ATC local anesthetics.

### 6.5. Hydrogels Loaded with Nanostructured Lipid Carrier

Nanostructured lipid carrier (NLC) are second-generation lipid nanomaterials [67] composed of a lipid component with solid and liquid lipids distributed in an aqueous emulsifier solution [68]. PENY is an antiepileptic medication; its noticeable stimulatory effect on connective tissue development suggests that it could be used topically as a wound-healing agent. However, it is not widely used due to its low solubility, bioavailability, and ineffective distribution during topical application. Motawea et al. [54] made an effort to sustain the release of PENY by making it into nanostructured lipid carriers (NLCs) and incorporating it into a hydrogel made up of CAR-934. The intermolecular hydrogen bond interaction between the PENY-NLC components evidenced the PENY entrapped inside the lipid matrix. After 48 h, the release of PENY from the hydrogel preparations was slower than their NLC. The obtained reduction in drug release results could be believed to be due to the polymer network of CAR-934. According to their results PENY-NLCs loaded in CAR-934 hydrogels could be novel, cost-efficient, and economically feasible carriers with significant potential in topical administration.

### 6.6. Hydrogels Loaded with Microemulsion

Tenoxicam (TEN) is an NSAID that is used to treat arthritis. However, long-term oral administration results in peptic ulcers. To overcome this adverse effect, Goindi S et al. [55] formulated a novel TEN-ME and incorporated it into a CAR-940 hydrogel for treating arthritis. The TEN-ME formulations produce higher levels of anti-inflammatory activity better than conventional topical dosage forms with effectiveness comparable to an oral formulation. Their research concluded that TEN-ME formulations could be a viable alternatives to the TEN oral formulations.

Res, 3,5,4′-trihydroxy-trans-stilbene (RTTS) is an effective treatment for osteoarthritis. Long-term oral administration of RTTS causes kidney damage. Because osteoarthritis is a topical disease, topical therapeutic delivery is much preferred. Hu et al. [57] developed RTTS-ME and incorporated it into CAR-940 hydrogel to treat osteoarthritis in a rabbit. Due to the decrease in particle size, the penetration of RTTS was more from ME and hydrogel formulations. It appears that papain-induced osteoarthritis in animals mimicked human osteoarthritis, as papain affects cartilage, but it did not interfere with the cartilage’s repair mechanism. Topical delivery of RTTS-ME incorporated in hydrogel noticeably relieved osteoarthritis signs by decreasing pro-inflammatory cytokines and improved macroscopic cartilage restoration. As a result, the hydrogel preparation could be an effective carrier for the topical administration of RTTS-ME to treat osteoarthritis.

### 6.7. Hydrogels Loaded with Nanoemulsion

Genistein (GEN) is an isoflavone that has recently received a lot of attention because of its effects on avoiding skin carcinoma and dermal aging on exposure to ultraviolet light. Due to its poor aqueous solubility, it cannot incorporate it into a topically applied form. To improve the solubility and permeability, Vargas et al. [56] created topical hydrogels encapsulated in GEN nanoemulsions. CAR-940-based hydrogel containing GEN-NE penetrates the skin in a sustained manner. They concluded that compared to octyldodecanol, there was detected a higher amount of GEN in the skin from the formulation composed of medium chain triglycerides (MCT) as the oily core. It is a promising delivery system for the skin.

### 6.8. Hydrogel Loaded with Cubosomes

The higher concentration of surfactant affects the size of the particle, entrapment efficiency of API, release from the formulation, and causes an adverse or toxic effect to the body. To overcome the above-mentioned drawbacks, Rapalli et al. [59] used the ‘Quality by Design (QbD) method to create a topical hydrogel comprising ketoconazole (KETO)-entrapped cubosomes with lesser surfactant concentrations. They were successful in their research, using the QbD method, by formulating the KETO-CUBO with a lower amount of surfactant than those reported in previous works of literature.

### 6.9. Hydrogel Loaded with Nanosponge

Nanosponge (NS) is a novel preparation that is a nanoporous carrier with a sponge-like network shaped by hyper cross-linking polymeric materials to form three-dimensional covalent structures used to incorporate nanoparticles with a non-collapsible and porous formation [69,70]. Clobetasol propionate (CP), a potent topical corticosteroid, possesses a high therapeutic potential for psoriasis. Meanwhile, common adverse effects, such as skin degeneration, steroidal acne, skin discoloration, and allergic skin reactions, limit its utility for topical administration. Kumar et al. [60] created a CP-NS based on these observations. They incorporated it into a CAR-934 hydrogel to reduce the adverse effects and control the CP release. They successfully prepared cyclodextrin-based CP-NS, with approximately 86% of CP released after 24 h. Incorporating CP-NS with CAR-934 hydrogel enhanced its suitability for topical administration. The anti-psoriatic potential of fabricated nanoformulation was further substantiated in vivo using a mouse tail model. Histological and biochemical findings showed appreciable anti-psoriatic activity of the prepared nanogel.

## 7. Conclusions

The formulations’ main goal is to deliver the APIs effectively, and to be toxicity-free and long-lasting. The novel formulations have benefits over traditional formulations, such as improved solubility, bioavailability, toxicity protection, improved pharmacological action, stability improvement, better tissue macrophage dispersion, sustained delivery, and protection from physiochemical deterioration. Incorporation of different APIs into novel formulations like nanoparticles, nanocapsules, liposomes, phytosomes, nanoemulsions, microemulsions, cubosomes, SLNP, NLC, nanosponge, and other novel formulations fulfill the above benefits.

Common skin diseases normally necessitate topical preparations to ensure patient compliance, while causing negligible systemic adverse effects. Treating skin diseases with a simple semi-solid dosage form is an appealing goal for dermatologists, patients, and pharmaceutical companies alike. The most significant challenge in achieving this goal is attaining adequate drug penetration, while reducing side effects. Numerous obstacles exist in the treatment of the skin on a topical basis. As an outcome, drug delivery through the skin is extremely complicated. Detailed physical and chemical properties and delivery systems are needed to estimate and analyze topical preparation and improve particle properties. In several cases, in vitro, ex vivo, and in vivo animal studies are the best method to study drug penetration and analyze the clinical efficiency and effectiveness of novel topical drugs delivery systems.

Hydrogels have been extensively used in topical applications because of their excellent biocompatibility, solubility in water, and three-dimensional pore structure that fits the extracellular matrix. Hydrogel research based on sources (mainly natural and synthetic polymers) has recently gained attraction that showed outstanding physicochemical properties that could be useful in treating a wide range of skin diseases. This innovation could allow for the localized delivery of APIs via topical applications to increase the action. There has been an increase in research on novel hydrogels over the last decade. Novel hydrogels were able to enhance the penetration of APIs, minimizing the risks of percutaneous absorption. These novel hydrogels have a strong pharmacological effect against different skin diseases. The combination of novel formulations with hydrogels offers a great opening to treat several currently available skin diseases. In this review, we learned that many researchers formulated novel formulations and a few did not conduct many characteristic studies (particle size, shape, entrapment efficacy, zeta potential, viscosity, pH, in-vitro release studies, etc.) for the prepared novel formulations. The novel formulation was then primarily mixed with hydrogel preparations. All of the researchers then carried out the evaluation studies for the novel hydrogel formulation by the studies. As evidenced by animal studies, many novel hydrogels demonstrated significant activity against various topical diseases and found this one to be stable, as evidenced by stability studies. There is a strong possibility that novel hydrogels based on natural and synthetic polymers will soon enter clinical trials and the market.

## Figures and Tables

**Table 1:** Novel formulation formulated with natural gelling agents, preparation methods, mixing with the hydrogels, and its use.

API	Novel Formulation	Novel Formulation Method	Gelling Agent for Making Hydrogels	Novel Formulation and Hydrogel Mixing Method	Use	References
Resveratrol	Liposomes	Film hydration method	Chitosan	Hand stirring method	Vaginal chlamydia infection	[32]
Vitamin C	Self-double-emulsifying drug delivery system	Two-step emulsification method	Xanthan gum	Mixing with a mechanical stirrer	Penetration enhance the skin	[33]
Rosmarinic acid	Nanoemulsions	Spontaneous emulsification method	Hydroxyethyl cellulose	Rosmarinic acid- nanoemulsion added to hydroxyethyl cellulose and stirred for 15 min	New anti-ageing skin products	[34]
Pentyl Gallate	Nanoemulsions	Spontaneous emulsification method	Chitosan	Chitosan added to pentyl gallate nanoemulsions	Increase skin penetration for herpis labialis	[35]
Phenytoin	Nanocapsule	Interfacial deposition	Chitosan	Chitosan was dispersed in the nanoemulsion and nanocapsule	Skin permeation and wound healing activity	[36]
	Nanoemulsion	Spontaneous emulsification method	Chitosan			
Ibuprofen	Microemulsion	NM	Xanthan gum	Mixing	To enhance percutaneous delivery	[37]
Terbinafine hydrochloride	Microemulsion	NM	Chitosan, and Natrosol 250	Gelling agent added to Terbinafine hydrochloride microemulsion	Anti fungal activity	[38]
Curcumin	Microemulsion	NM	Xanthan and galactomannan	Curcumin microemulsion was added to hydrogel preparation	To increase skin penetration and anti-inflammatory activity	[39]
Baicalin	Nanocrystals	Coupling homogenization technology followed by spray-drying technology	Hyaluronic acid	Baicalin nanocrystals was added into hyaluronic acid hydrogel and mixed	To improve skin permeation	[40]
Silver sulfadiazine	Cubosome	Emulsification method	Chitosan	Cubosome incorporated into chitosan	Increasing skin permeation and for treating topical burn	[41]

NM—Not mentioned by the researcher.

**Table 2:** Different novel formulation evaluation studies before incorporating into hydrogel.

API-Novel Formulation	Droplet/Particle Range	Drug Content/Entrapment Efficiency (%)	PDI	Zeta Potential (mV)	pH	Viscosity	Shape	Time	DR (%)	References
RVT-LS	100 to 200 nm (Average 158 ± 22 nm)	85 ± 2	0.077	−6.72 ± 2.47	NP	appropriate viscosity (NM)	Spherical	8 h	61	[32]
Vit C-SDEDDS	0.06 to 60.26 μm (Average 17.13 ± 2.50 μm)	NP	NP	NP	NP	NP	NP	NP	NP	[33]
ROS-NE	180.57 ± 1.82 to 224.67 ± 2.31 nm	98.59 ± 2.12 to 107.69 ± 6.28	0.123 ± 0.021 to 0.230 ± 0.036	−39.65 ± 1.53 to 46.17 ± 3.90	3.85 ± 0.07 to 4.73 ± 0.07	1.1 to 1.3 cps	NP	8 h	71.8 ± 1.98	[34]
PG-NE	164.3 ± 7.4 nm	96.2 ± 3.4	0.12 ± 0.03	−48.9 ± 2.1	5.5 ± 0.2	Near to 1 cps	NM	NP	NP	[35]
PENY-NC	161 ± 4 nm	95.2 ± 1.4	0.14 ± 0.01	−15.7 ± 0.3	5.6 ± 0.1	NP	Spherical	3 h 24 h	27 ± 1 67 ± 1	[36]
PENY-NE	125 ± 6 nm	88.7 ± 1.1	0.12 ± 0.01	−10.8 ± 0.4	5.0 ± 0.7	NP	Spherical	3 h 24 h	36 ± 2 77 ± 2	[36]
IBU-ME	14.34 ± 0.98 nm	NP	0.220 ± 0.075	NP	5.23	0.2025 ± 0.003 Pas	NP	12 h	46.78 ± 4.59	[37]
TER-ME	44.98 ± 27.34 nm	NP	NP	NP	NP	77.98 ± 0.75 cp	Spherical	NP	NP	[38]
CUR-ME	231.8 ± 7.6 nm	99.50	NP	NP	NP	NP	NP	NP	NP	[39]
BCN-NCY	189.21 ± 0.36 nm	NP	NP	NP	NP	NP	Spherical Gel TEM showed network structure	4 h	65.3 ± 3.2	[40]
SSD-CUBO	152.3 to 389.6 nm	86.05 ± 3.86 to 94.56 ± 1.40	0.25 ± 0.004 to 0.65 ± 0.45	NP	NP	NP	Cubic	NP	NP	[41]

NP: Not performed by the researchers; NM: Not mentioned by the researchers.

**Table 3:** Physical evaluation studies for novel hydrogel formulation prepared with natural gelling agent.

API-Novel Formulation	Hydrogel Made of	Hydrogel Concentration	Formulation Concentration	Loading Efficient (%)	Ph	Droplet SIZE (nm)	Zeta Potential	Viscosity	SEM	References
RVT-LS	CHI	2.5% *w*/*w*	20% *w*/*w*	NP	NP	NP	NP	NP	NP	[32]
Vit-C-SEDDS	XG	2%	5%	NP	5.5 ± 0.1	NP	NP	4.62 ± 0.50	The structure of Vit-C was completely destroyed in freeze-dried hydrogel	[33]
ROS-NE	HEC	1% *w*/*v*	0.1% *w*/*v*	98.50 ± 3.59 to 100.79 ± 1.98	3.83 ± 0.05 to 4.73 ± 0.07	NP	NP	NP	NP	[34]
PG-NE	CHI	2.5% *w*/*w*	0.5% *w*/*w*	94.4 ± 4.8	5.0 ± 0.3	297.0 ± 8.6	52.6 ± 0.1	NP	NP	[35]
PENY-NCY	CHI	2.75% *w*/*v*	0.025% *w*/*v*	0.24 ± 0.01 mg/gm	4.8 ± 0.1	NP	NP	24.23 ± 2.70 pasn	NP	[36]
PENY-NE	CHI	2.75% *w*/*v*	0.025% *w*/*v*	0.25 ± 0.01 mg/gm	4.7 ± 0.2	NP	NP	24.53 ± 3.71 pasn	NP	[36]
IBU-ME	XG	0.25–1%	5%	NP	NP	5.17 ± 0.01	NP	1.12 ± 0.15 to 6.80 ± 0.02	NP	[37]
TER-ME	CHI, NAT, and CAR	CHI-1%,	1%	NP	3.04 ± 0.02	NP	NP	5044.03 ± 22.43	NP	[38]
		NAT-4%,								
		CAR-1%								
CUR-ME	X-GAL	1.25%	NP	103.90	5.3	NP	NP	NP	Network structure	[39]
BCN-NCY	HA	0.5%, 1%, 1.5% and 2%, *w*/*v*	1%	NP	NP	NP	NP	NP	Porous structure	[40]
SSD-CUBO	CHI	NP	NP	NP	4	NP	NP	NP	NP	[41]

NP: Not performed by the researchers.

**Table 4:** Novel natural hydrogel formulation in vitro, in vivo evaluation studies details.

API-Formulation-Hydrogel	In Vitro Release Study		In Vitro Kinetics		In Vivo Skin Studies			Animal Used	References
	Time	Drug Released (%)	Model	Mechanism	Model Skin	Time	DR		
RVT-LS-CHI	8 h	38	NP	NP	NP	NP	NP	NP	[32]
Vit C-SEDDS-XG	6 h	72.33	Weibull model	Fickian diffusion and Case-II transport	Porcine abdominal skin	12 h	12%	NP	[33]
ROS-NE-HEC	8 h	57 ± 0.36	NP	NP	Pig ear skin	8 h	0.65 ± 0.08 µg/cm^2^	NP	[34]
PG-NE-CHI	24 h	Not shown	NP	NP	Porcine ear skin	NP	NP	NP	[35]
PENY-NC-CHI	12 h	43 ± 1	NP	NP	Porcine ear skin	12 h	NP	Male Wistar rats	[36]
PENY-NE-CHI	12 h	53 ± 1	NP	NP	Porcine ear skin	12 h	NP	Male Wistar rats	[36]
IBU-ME-XG	12	ME-XG-H1-23%, ME-XG-H2-16%, ME-XG-H3-14%, and ME-XG-4-11%	Zero order	NP	NP	NP	NP	Male Wistar rats	[37]
TER-ME-CHI TER-ME-NAT	7 h	8.70	Zero order	NP	NP	NP	NP	NP	[38]
CUR-ME-X-GAL	10 h	<60	Higuchi	Diffusion controlled	Porcine ear skin	NP	NP	NP	[39]
BCN-NCY-HA	6 h	0.5 and 1% CAR->95% 1.5%CAR-85.4% 2%CAR-72.3%	NP	NP	Mouse abdominal skin	12 h	NM	NP	[40]
SSD-CUBO-CHI	NP	NP	Zero order	Diffusion controlled	NP	NP	NP	NP	[41]

NP: Not performed by the researchers; NM: Not mentioned by the researchers.

**Table 5:** Novel formulation formulated with synthetic gelling agents, preparation methods, mixing with the hydrogels, and its use.

API	Novel Formulation	Novel Formulation Method	Gelling Agent for Making Hydrogels	Formulation and Hydrogel Mixing Method	Use	References
IR780 iodide and IR792 perchlorate	LP	Thin-film hydration method	Poloxamer 407, and 188	Gelling agents added to novel formulation	Targeted tumor photothermal therapy	[51]
Buparvaquone	SNDDS	NM	Carbopol 940	Carbopol was mixed with novel formulation	Cutaneous leishmaniasis	[52]
Escin and escin β-sitosterol phytosome	PHY	NM	Carbopol 934	Hydrogel added dropwise to the novel formulation	Antihyperalgesic activity	[53]
Pentyl Gallate	NE	Spontaneous emulsification method	Aristoflex AVC	Gelling agent added to novel formulation	Increase skin penetration for herpis labialis	[35]
Simvastatin	MP	Ionic gelation method	Poly vinyl alcohol	Chemical cross linking method	Sustained SIM release and wound healing activity	[44]
5,10,15,20-tetrakis(1-methylpyridinium-4-yl)-porphyrin tetra-iodide	NP	Solvent evaporation method	Carbopol-940	Novel formulation added to CAR-940	Photodynamic applications	[31]
Phenytoin	NLC	Hot homogenization followed by ultrasonication method	Carbomer 934	CAR dispersed in the NLC suspension	Increasing the entrapment efficacy and to sustained release.	[54]
Tenoxicam	ME	NM	Carbopol 940	TEN-ME gelled with CAR-940	Arthritis	[55]
Genistein	NE	Spontaneous emulsification process	Carbopol 940	Hand stirring method	To enhance skin permeation	[56]
Terbinafine hydrochloride	ME	NM	Carbopol 974	Mixing in magnetic stirrer	Anti fungal activity	[38]
Silver sulfadiazine	CUBO	Emulsification method	Carbomer 934	Cubosome incorporated into CAR-934	For improving skin permeation and to treat topical burn	[41]
Resveratrol 3,5,4′-trihydroxy-trans-stilbene	ME	NM	Carbopol 940	CAR dispersed in novel formulation	Treatment of osteoarthritis	[57]
Articaine	NC and SLNP	NM	Aristoflex AVC	NC and SLNP was incorporated into ART hydrogel	In-vitro release studies	[58]
ketoconazole	CUBO	Hot emulsification method	Carbopol 971P	CUBO added to CAR-971P hydrogel and stirred (350 rpm)	In-vitro release and ex vivo penetration studies	[59]
Clobetasol propionate	NS	NM	Carbopol 934	CP-NS incorporated into CAR-934 hydrogel	Anti-psoriatic studies	[60]

NM: Not mentioned by the researchers.

**Table 6:** Different novel formulation evaluation studies before incorporating into hydrogel.

API-Novel Formulation	Droplet/Particle Range (nm)	Entrapment Efficiency (%)	PDI	Zeta Potential (mV)	pH	Viscosity	Shape	SEM	DR			References
									Time	%	Kinetics	
IR 780-LS	Around 130	NP	0.185	NP	NP	NP	Spherical	Freeze dried formulation-porous sponge-like structures	NP	NP	NP	[51]
IR 792-LS	122	NP	NP	NP	NP	NP	Spherical	NP	NP	NP	NP	
BQ-SEDDS	255 ± 37	NP	0.685 ± 0.085	−13.5 ± 0.2	NP	NP	Spherical	NP	NP	NP	NP	[52]
SIM-MP	between 0.5 μm <10 μm	51 ± 0.7 to 82 ± 0.3	NP	NP	NP	NP	Spherical	Rough	8 h	3.8 ± 1.1 to 9.9 ± 0.4	NP	[44]
TMPTI-NPT	118 ± 5 and 133 ± 2	55.8 ± 1.1 to 92.5 ± 3.5	0.17 ± 0.01 to 0.18 ± 0.03	−21.6 ± 1.0 to 26.7 ± 3.0	NP	NP	Spherical	NP	NP	NP	NP	[31]
PENY-NLC	121.45 ± 2.65 to 258.24 ± 6.59	55.24 ± 1.60 to 88.80 ± 4.13	0.18 ± 0.01 to 0.41 ± 0.02	−15.44 ± 0.87 to −32.26 ± 1.68	5.67 ± 0.02 to 6.49 ± 0.23	NP	Spherical	Smooth surface	48 h	73.47 ± 2.45	Higuchi model	[54]
TEN-ME	106 to 122	99	NP	Near zero	5.5 to 5.7	11,100 to 12,000 cps	Spherical	NP	NP	NP	NP	[55]
GEN-NE	GEN-NE-MCT-240 ± 28	93.00 ± 2.00	<0.25	−37 ± 4	5.8 ± 0.3	1.50 ± 0.10	NP	NP	NP	NP	NP	[56]
	GEN-NE-ODD-247 ± 23	96.00 ± 1.00	<0.25	−36 ± 4	5.9 ± 0.2	1.80 ± 0.07	NP	NP	NP	NP	NP	
SSD-CUBO	150 to 400	74.93 ± 0.903 to 92.10 ± 0.250	0.25 ± 0.004 to 0.65 ± 0.45	−17.61 to 7.41	NP	NP	Cubic	NP	NP	NP	Zero order	[41]
RTTS-ME	17.5 ± 1.4	NP	0.068 ± 0.016	−11.8 ± 0.5	NP	14.2 ± 0.1 mPa s	Spherical	NP	NP	NP	NP	[57]
ART-NC	4455 ± 21	78.10	0068 ± 0005	NP	8.1 ± 1.2	NP	Spherical	Smooth surface	400 min	50	Higuchi model	[58]
ART-SLNP	2499 ± 22	65.70	0113 ± 0008	NP	7.9 ± 0.9	NP	Spherical	Smooth surface	300 min	50	NP	
KETO-CUBO	188.6 ± 5.992 to 381 ± 2.082	15.79 ± 1.23 to 72.22 ± 1.08	0.437 ± 0.032 to 0.918 ± 0.06	NP	NP	NP	Cubic	NP	24 h	67	Korsmeyer–Peppas model	[59]
CP-NS	194.27 ± 49.24 nm	56.33 ± 0.94%	0.498 ± 0.095	−21.83 ± 0.95	NP	NP	Porus and crystalline nature	Freeze dried formulation-porous sponge-like structures.	1st h 6th h 24th h	32.39 ± 0.10 55.81 ± 0.60 86.25 ± 0.28	Higuchi model	[60]

NP: Not performed by the researchers; NM: Not mentioned by the researchers.

**Table 7:** Physical evaluation studies for novel hydrogel formulation prepared with synthetic gelling agent.

API-Novel Formulation	Hydrogel Made of	Hydrogel Concentration	Formulation Concentration	Particle Size	PDI	ZP	Loading Efficient (%)	pH	Viscosity	References
IR 780-LS	Pol-407 and 188	NM	NM	NP	NP	NP	NP	NP	NP	[51]
Escin and escin β-sitosterol PHY	CAR-934	1%	1–5%	NP	NP	NP	NP	4.95 to 6.3	1.0 ± 0.4 to 31.7 ± 0.5 Pas	[53]
BQ-SNDDS	CAR-940	1%	2%	266 ± 99	0.609 ± 0.046	−28.7 ± 1.1	NP	NP	Appropriate for skin application (NM)	[52]
BQ-SNDDS	CAR-940	2%	2%	260 ± 35	0.758 ± 0.072	−34.5 ± 1.2	NP	NP	Appropriate for skin application (NM)	
SIM-MP	PVA	5, 7 and 9% *w*/*v*	2.5, 5, and 10 mg	NP	NP	NP	NP	NP	NP	[44]
TMPTI-NPT	CAR-940	NM	NM	NP	NP	NP	NP	5.7 to 6.6	NP	[31]
PENY-NLC	CAR-934	1% *w*/*v*	0.05%	NP	NP	NP	90 to 100	6.88 ± 0.30 and 7.27 ± 0.16	16 to 18 ps	[54]
TEN-ME	CAR-940	NM	NM	NP	NP	NP	NP	NP	NP	[55]
GEN-NE-MCT	CAR-940	0.5%	0.1 (1 mg/gm)	NP	NP	NP	92.00 ± 3.00	7	25–33 cP	[56]
GEN-NE-ODD	CAR-940	0.5%	0.1 (1 mg/gm)	NP	NP	NP	91.00 ± 6.00	7	58–64 cP	
TER-ME	CAR-940	1%	NP	NP	NP	NP	NP	3.04 ± 0.02	5044.03 ± 22.43	[38]
SSD-CUBO	CAR-934	0.5, 1, 1.5 and 2%	NP	NP	NP		76 to 91	8	925 to 982 cps at 10 rpm	[41]
RTTS-ME	CAR-940	1.50%	2%	NP	NP	NP	NP	6.7	171.1 ± 0.3 mPa·s	[57]
ATC-NC	ART	2%	20 mg/gm	463.2 ± 24.7 nm	0.190 ± 0.013	NP	NP	NP	19,554.99 Pa·s^−1^	[58]
ATC-SLNP	ART	2%	20 mg/gm	315.3 ± 20.1 nm	0.206 ± 0.009	NP	NP	NP	22,090.23 Pa·s^−1^	
PG-NE	ART	2% *w*/*w*	0.5% *w*/*w*	97.3 ± 2.7	5.1 ± 0.2	162.1 ± 1.1	−46.5 ± 1.3	NP	NP	[35]
KETO-CUBO	CAR-971P	1% *w*/*w*	0.2% *w*/*w*	NP	NP	NP	96.81 ± 4.50	NP	25,586.67 ± 743.32 at 1.0 rpm	[59]
CP-NS	CAR-934	NP	NP	NP	NP	NP	NP	NP	NP	[60]

NP: Not performed by the researchers; NM: Not mentioned by the researchers.

**Table 8:** Novel synthetic hydrogel formulation in vitro, in vivo evaluation studies details.

API-Novel Formulation-Hydrogel	In Vitro Release Study				Ex Vivo Skin Studies					Animal Used	References
	Time	Drug Released (%)	Kinetics	Mechanism	Model Skin	Time	DR (%)	Kinetics	Mechanism		
IR 780-LS-POL	12 h	>90	NP	NP	NP	NP	NP	NP	NP	CT-26 cancer bearing mice	[51]
BQ-SNDDS-CAR-940	NP	NP	NP	NP	BALB/c mouse skin	NP	NP	Zero order	Case II drug transport	BALB/c mouse	[52]
Escin and ES-PHY-CAR-934	NP	NP	NP	NP	NP	NP	NP	NP	NP	Wistar rat	[53]
SIM-MP-PVA	7 days	2.5 mg SIM-92% 5 mg SIM-60% 10 mg SIM-36%	NP	NP	NP	NP	NP	NP	NP	Wistar rat	[44]
TMPTI-NPT-CAR-940	4.5 h 24 h	20 40	Korsmeyer–Peppas model	Non-fickian diffusion	Porcine skin	24 h	Not detected	NP	NP	NP	[31]
PENY-NLC-CAR-934	48 h	51.13 ± 1.69	Korsmeyer–Peppas model	Non-fickian diffusion	NP	NP	NP	NP	NP	NP	[54]
TEN-ME-CAR-940	NP	NP	NP	NP	Laca mouse skin	24 h	64–71	NP	NP	Rat Sprague-Dawley	[55]
GEN-NE-MCT-CAR-940	8 h	NP	NP	NP	Porcine skin	In 8 h	100 µg/cm^2^	NP	NP	NP	[56]
GEN-NE-ODD-CAR-940	8 h	NP	NP	NP	Porcine skin	In 8 h	150 µg/cm^2^	NP	NP	NP	
SSD-CUBO-CAR-934	12 h	76 to 98	NP	NP	NP	NP	NP	NP	NP	Male adult Wister rats	[41]
RTTS-ME-CAR-940	NP	NP	NP	NP	Porcine abdominal skin	NP	NP	NP	NP	Rabbit	[57]
ATC-NC-ART	8 h	NP	Higuchi	Diffusion	NP	NP	NP	NP	NP	NP	[58]
ATC-SLNP-ART	NP	NP	NP	NP	NP	NP	NP	NP	NP	NP	
PG-NE	24	Not shown	NP	NP	Porcine ear skin	NP	NP	NP	NP	NP	[35]
KETO-CUBO-CAR-971P	NP	NP	NP	NP	Goat skin	24 h	92.73	NP	NP	NP	[59]
CP-NS-CAR-934	NP	NP	NP	NP	NP	NP	NP	NP	NP	Male Swiss albino mice	[60]

NP: Not performed by the researchers.

## Abbreviations

ALG	Alginate
API	Active pharmaceutical ingredients
ATC	Articaine
BCN	Baicalin
BQ	Buparvaquone
Ca	Calcium
CAR	Carbopol
CHI	Chitosan
CL	Cutaneous leishmaniasis
CP	Clobetasol propionate
CUBO	Cubosomes
CUR	Curcumin
ES	Escin-sitosterol
GAL	Galactomannan
GEN	Genistein
HA	Hyaluronic acid
IBU	Ibuprofen
KETO	Ketoconazole
LS	Liposomes
MP	Microparticle
NC	Nanocapsule
NCY	Nanocrystals
NE	Nanoemulsions
NLC	Nanostructured lipid carrier
NM	Not mentioned
NP	Not performed
NPT	Nanoparticle
NS	Nanosponge
PENY	Phenytoin
PG	Pentyl Gallate
PHY	Phytosomes
POL	Poloxamer
PVA	Polyvinyl alcohol
QbD	Quality by Design
ROS	Rosmarinic acid
RTTS	Res, 3,5,4′-trihydroxy-trans-stilbene
RVT	Resveratrol
SC	Stratum corneum
SDEDDS	Self-double-emulsifying drug delivery system
SIM	Simvastatin
SLNP	Solid lipid nanoparticles
SNEDDS	Self-nanoemulsifying drug delivery systems
SSD	Silver sulfadiazine
SSDF	Semi-solid dosage form
TDDS	Topical drug delivery system
TEN	Tenoxicam
TER	Terbinafine hydrochloride
TMPTI	5,10,15,20-tetrakis(1-methylpyridinium-4-yl)-porphyrin tetra-iodide
X	Xanthan
XG	Xanthan gum
ZnO	Zinc oxide
